# Supplementary figures and images for: The effect of nature exposure on pain experience and quality of life in patients with chronic pain: A systematic review and meta-analysis protocol
Source: PLoS One. 2023 Sep 28;18(9):e0291053. doi: 10.1371/journal.pone.0291053 (PMC10538778; doi:10.1371/journal.pone.0291053)

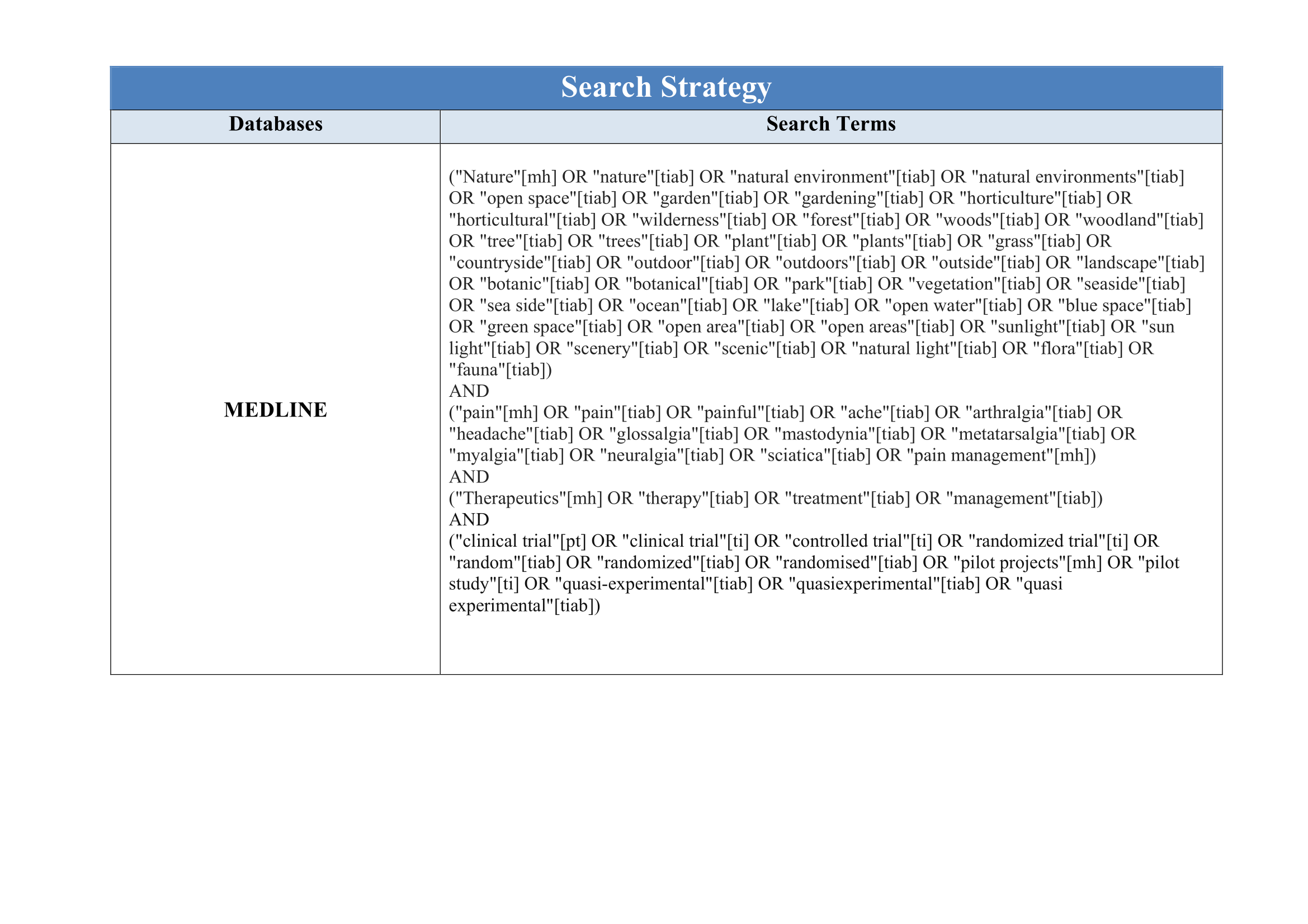

Supplement: S1 Table — (TIFF) [file pone.0291053.s001.tiff]
