# Supplementary material for: The effect of nature exposure on pain experience and quality of life in patients with chronic pain: A systematic review and meta-analysis protocol
Source: PLoS One. 2023 Sep 28;18(9):e0291053. doi: 10.1371/journal.pone.0291053 (PMC10538778; doi:10.1371/journal.pone.0291053)
Supplement: S1 Appendix — (DOC) [file pone.0291053.s002.doc]

**PRISMA-P (Preferred Reporting Items for Systematic review and Meta-Analysis Protocols) 2015 checklist: recommended items to address in a systematic review protocol***

| Section and topic | Item No | Checklist item |
| --- | --- | --- |
| ADMINISTRATIVE INFORMATION | | |
| Title: |  |  |
| Identification | 1a | Identify the report as a protocol of a systematic review PAGE 1, LINE 4 |
| Update | 1b | If the protocol is for an update of a previous systematic review, identify as such. Not Applicable |
| Registration | 2 | If registered, provide the name of the registry (such as PROSPERO) and registration number systematic review was registered on PROSPERO (ID: CRD42021226949) |
| Authors: |  |  |
| Contact | 3a | Provide name, institutional affiliation, e-mail address of all protocol authors; provide physical mailing address of corresponding author. PAGE 1, LINES 9-12 |
| Contributions | 3b | Describe contributions of protocol authors and identify the guarantor of the review PAGE 1, LINES 17; PAGE 14, LINES 293-299 |
| Amendments | 4 | If the protocol represents an amendment of a previously completed or published protocol, identify as such and list changes; otherwise, state plan for documenting important protocol amendments Not applicable |
| Support: |  |  |
| Sources | 5a | Indicate sources of financial or other support for the review Not Applicable |
| Sponsor | 5b | Provide name for the review funder and/or sponsor Not Applicable |
| Role of sponsor or funder | 5c | Describe roles of funder(s), sponsor(s), and/or institution(s), if any, in developing the protocol Not Applicable |
| INTRODUCTION | | |
| Rationale | 6 | Describe the rationale for the review in the context of what is already known. PAGE 3, LINES 46-68; PAGE 4, LINES 69-82 |
| Objectives | 7 | Provide an explicit statement of the question(s) the review will address with reference to participants, interventions, comparators, and outcomes (PICO) PAGE 4, LINES 83-96 |
| METHODS | | |
| Eligibility criteria | 8 | Specify the study characteristics (such as PICO, study design, setting, time frame) and report characteristics (such as years considered, language, publication status) to be used as criteria for eligibility for the review PAGE 5/6, LINES 96-115 |
| Information sources | 9 | Describe all intended information sources (such as electronic databases, contact with study authors, trial registers or other grey literature sources) with planned dates of coverage PAGE 6, LINES 117-121 |
| Search strategy | 10 | Present draft of search strategy to be used for at least one electronic database, including planned limits, such that it could be repeated PAGE 6, LINES 117-123; Table 1 |
| Study records: |  |  |
| Data management | 11a | Describe the mechanism(s) that will be used to manage records and data throughout the review PAGE 7, LINES 134-135 |
| Selection process | 11b | State the process that will be used for selecting studies (such as two independent reviewers) through each phase of the review (that is, screening, eligibility and inclusion in meta-analysis) PAGE 6, LINES 126-132 |
| Data collection process | 11c | Describe planned method of extracting data from reports (such as piloting forms, done independently, in duplicate), any processes for obtaining and confirming data from investigators PAGE 7, LINES 134-136 |
| Data items | 12 | List and define all variables for which data will be sought (such as PICO items, funding sources), any pre-planned data assumptions and simplifications PAGE 7, LINES 137-144 |
| Outcomes and prioritization | 13 | List and define all outcomes for which data will be sought, including prioritization of main and additional outcomes, with rationale. PAGE 7, LINES 147-155 |
| Risk of bias in individual studies | 14 | Describe anticipated methods for assessing risk of bias of individual studies, including whether this will be done at the outcome or study level, or both; state how this information will be used in data synthesis PAGE 8, LINES 157-163 |
| Data synthesis | 15a | Describe criteria under which study data will be quantitatively synthesised PAGE 8, LINES 166-168 |
|  | 15b | If data are appropriate for quantitative synthesis, describe planned summary measures, methods of handling data and methods of combining data from studies, including any planned exploration of consistency (such as I^2^, Kendall’s τ) PAGE 8/9, LINES 168-181 |
|  | 15c | Describe any proposed additional analyses (such as sensitivity or subgroup analyses, meta-regression) PAGES 9, LINES 182-189 |
|  | 15d | If quantitative synthesis is not appropriate, describe the type of summary planned PAGE 9, LINES 189-192 |
| Meta-bias(es) | 16 | Specify any planned assessment of meta-bias(es) (such as publication bias across studies, selective reporting within studies) PAGE 9, LINES 188-189 |
| Confidence in cumulative evidence | 17 | Describe how the strength of the body of evidence will be assessed (such as GRADE) PAGE 9/10, LINES 193-203 |

*** It is strongly recommended that this checklist be read in conjunction with the PRISMA-P Explanation and Elaboration (cite when available) for important clarification on the items. Amendments to a review protocol should be tracked and dated. The copyright for PRISMA-P (including checklist) is held by the PRISMA-P Group and is distributed under a Creative Commons Attribution Licence 4.0.**

*From: Shamseer L, Moher D, Clarke M, Ghersi D, Liberati A, Petticrew M, Shekelle P, Stewart L, PRISMA-P Group. Preferred reporting items for systematic review and meta-analysis protocols (PRISMA-P) 2015: elaboration and explanation. BMJ. 2015 Jan 2;349(jan02 1):g7647.*
