# Supplementary material for: The effect of nature exposure on pain experience and quality of life in patients with chronic pain: A systematic review and meta-analysis protocol
Source: PLoS One. 2023 Sep 28;18(9):e0291053. doi: 10.1371/journal.pone.0291053 (PMC10538778; doi:10.1371/journal.pone.0291053)
Supplement: S2 Appendix — (DOCX) [file pone.0291053.s003.docx]

| Search Strategy | |
| --- | --- |
| Databases | **Search Terms** |
| MEDLINE | ("Nature"[mh] OR "nature"[tiab] OR "natural environment"[tiab] OR "natural environments"[tiab] OR "open space"[tiab] OR "garden"[tiab] OR "gardening"[tiab] OR "horticulture"[tiab] OR "horticultural"[tiab] OR "wilderness"[tiab] OR "forest"[tiab] OR "woods"[tiab] OR "woodland"[tiab] OR "tree"[tiab] OR "trees"[tiab] OR "plant"[tiab] OR "plants"[tiab] OR "grass"[tiab] OR "countryside"[tiab] OR "outdoor"[tiab] OR "outdoors"[tiab] OR "outside"[tiab] OR "landscape"[tiab] OR "botanic"[tiab] OR "botanical"[tiab] OR "park"[tiab] OR "vegetation"[tiab] OR "seaside"[tiab] OR "sea side"[tiab] OR "ocean"[tiab] OR "lake"[tiab] OR "open water"[tiab] OR "blue space"[tiab] OR "green space"[tiab] OR "open area"[tiab] OR "open areas"[tiab] OR "sunlight"[tiab] OR "sun light"[tiab] OR "scenery"[tiab] OR "scenic"[tiab] OR "natural light"[tiab] OR "flora"[tiab] OR "fauna"[tiab])  AND  ("pain"[mh] OR "pain"[tiab] OR "painful"[tiab] OR "ache"[tiab] OR "arthralgia"[tiab] OR "headache"[tiab] OR "glossalgia"[tiab] OR "mastodynia"[tiab] OR "metatarsalgia"[tiab] OR "myalgia"[tiab] OR "neuralgia"[tiab] OR "sciatica"[tiab] OR "pain management"[mh])  AND  ("Therapeutics"[mh] OR "therapy"[tiab] OR "treatment"[tiab] OR "management"[tiab])  AND  ("clinical trial"[pt] OR "clinical trial"[ti] OR "controlled trial"[ti] OR "randomized trial"[ti] OR "random"[tiab] OR "randomized"[tiab] OR "randomised"[tiab] OR "pilot projects"[mh] OR "pilot study"[ti] OR "quasi-experimental"[tiab] OR "quasiexperimental"[tiab] OR "quasi experimental"[tiab]) |
| Embase | ('nature':ti,ab OR 'natural environment':ti,ab OR 'natural environments':ti,ab OR 'open space':ti,ab OR 'garden':ti,ab OR 'gardening':ti,ab OR 'horticulture'/exp OR 'horticulture':ti,ab OR 'horticultural':ti,ab OR 'wilderness'/exp OR 'wilderness':ti,ab OR 'forest'/exp OR 'forest':ti,ab OR 'woods':ti,ab OR 'woodland':ti,ab OR 'tree'/de OR 'tree':ti,ab OR 'trees':ti,ab OR 'plant'/de OR 'plant':ti,ab OR 'plants':ti,ab OR 'grass'/de OR 'grass':ti,ab OR 'countryside':ti,ab OR 'outdoor':ti,ab OR 'outdoors':ti,ab OR 'outside':ti,ab OR 'landscape'/exp OR 'landscape':ti,ab OR 'botanic':ti,ab OR 'botanical':ti,ab OR 'park':ti,ab OR 'vegetation'/exp OR 'vegetation':ti,ab OR 'seashore'/exp OR 'seaside':ti,ab OR 'sea side':ti,ab OR 'sea'/exp OR 'ocean':ti,ab OR 'lake'/exp OR 'lake':ti,ab OR 'open water':ti,ab OR 'blue space':ti,ab OR 'green space':ti,ab OR 'open area':ti,ab OR 'open areas':ti,ab OR 'sunlight'/exp OR 'sunlight':ti,ab OR 'sun light':ti,ab OR 'scenery':ti,ab OR 'scenic':ti,ab OR 'natural light':ti,ab OR 'flora'/de OR 'flora':ti,ab OR 'fauna'/de OR 'fauna':ti,ab)  AND  ('pain'/exp OR 'pain':ti,ab OR 'painful':ti,ab OR 'ache':ti,ab OR 'arthralgia'/exp OR 'arthralgia':ti,ab OR 'earache':ti,ab OR 'toothache':ti,ab OR 'headache':ti,ab OR 'glossalgia':ti,ab OR 'mastodynia':ti,ab OR 'metatarsalgia':ti,ab OR 'myalgia'/exp OR 'myalgia':ti,ab OR 'neuralgia'/exp OR 'neuralgia':ti,ab OR 'sciatica'/exp OR 'sciatica':ti,ab)  AND  ('therapy'/exp OR 'therapy':ti,ab OR 'treatment':ti,ab OR 'management':ti,ab |
| CINAHL | (MH "Natural environment" OR TI "nature" OR AB "nature" OR TI "natural environment" OR AB "natural environment" OR TI "natural environments" OR AB "natural environments" OR TI "open space" OR AB "open space" OR TI "garden" OR AB "garden" OR TI "gardening" OR AB "gardening" OR TI "horticulture" OR AB "horticulture" OR TI "horticultural" OR AB "horticultural" OR TI "wilderness" OR AB "wilderness" OR TI "forest" OR AB "forest" OR TI "woods" OR AB "woods" OR TI "woodland" OR AB "woodland" OR TI "tree" OR AB "tree" OR TI "trees" OR AB "trees" OR TI "plant" OR AB "plant" OR TI "plants" OR AB "plants" OR TI "grass" OR AB "grass" OR TI "countryside" OR AB "countryside" OR TI "outdoor" OR AB "outdoor" OR TI "outdoors" OR AB "outdoors" OR TI "outside" OR AB "outside" OR TI "landscape" OR AB "landscape" OR TI "botanic" OR AB "botanic" OR TI "botanical" OR AB "botanical" OR TI "park" OR AB "park" OR TI "vegetation" OR AB "vegetation" OR TI "seaside" OR AB "seaside" OR TI "sea side" OR AB "sea side" OR TI "ocean" OR AB "ocean" OR TI "lake" OR AB "lake" OR TI "open water" OR AB "open  water" OR TI "blue space" OR AB "blue space" OR TI "green space" OR AB "green space" OR TI "open area" OR AB "open area" OR TI "open areas" OR AB "open areas" OR TI "sunlight" OR AB "sunlight" OR TI "sun light" OR AB "sun light" OR TI "scenery" OR AB "scenery" OR TI "scenic" OR AB "scenic" OR TI "natural light" OR AB "natural light" OR TI "flora" OR AB "flora" OR TI "fauna" OR AB "fauna")  AND  (MH "pain" OR TI "pain" OR AB "pain" OR TI "painful" OR AB "painful" OR TI "ache" OR AB "ache" OR TI "arthralgia" OR AB "arthralgia" OR TI "earache" OR AB "earache" OR TI "toothache" OR AB "toothache" OR TI "headache" OR AB "headache" OR TI "glossalgia" OR AB "glossalgia" OR TI "mastodynia" OR AB "mastodynia" OR TI "metatarsalgia" OR AB "metatarsalgia" OR TI "myalgia" OR AB "myalgia" OR TI "neuralgia" OR AB "neuralgia" OR TI "sciatica" OR AB "sciatica" OR MH "pain management")  AND  (MH "Therapeutics" OR TI "therapy" OR AB "therapy" OR TI "treatment" OR AB "treatment" OR TI "management" OR AB "management" |
