# Supplementary material for: The effect of nature exposure on pain experience and quality of life in patients with chronic pain: A systematic review and meta-analysis protocol
Source: PLoS One. 2023 Sep 28;18(9):e0291053. doi: 10.1371/journal.pone.0291053 (PMC10538778; doi:10.1371/journal.pone.0291053)
Supplement: Attachment — Submitted filename: Response to Reviewers.pdf [file pone.0291053.s004.pdf]

Hackensack Meridian
School of Medicine

Dear Editor and Reviewers,

Thank you for your comments and feedback on our manuscript entitled "The Effect of Nature Exposure on Pain Experience and Quality of Life in Patients with Chronic Pain: A Systematic Review and Meta-Analysis Protocol." We appreciate you taking the time to help improve our systematic review protocol. We have spent much time improving the methodology and organization of our protocol. Our revisions are marked in red in the updated manuscript.

Below are our responses to the following comments provided by the Editor:

Point 1: Please note that there is a difference between methodology guidelines and reporting guidelines. The PRISMA-P is rather a reporting guideline. The authors should refer to adequate methodology guidelines for the conduct of this systematic review (in this particular case, the guidelines in the Cochrane handbook may be appropriate), then to the PRISMA-P as a reporting guideline for this protocol manuscript. Therefore, the sentence "This systematic review protocol is designed in accordance with the guidelines provided in the Preferred Reporting Items for Systematic Reviews and Meta-Analysis Protocols (PRISMA-P) statement" should be amended.

Response 1: We thank you for bringing this to our attention and we apologize for incorrectly using the term "methodology guidelines" in regards to the PRISMA-P. We changed the sentence to acknowledge that the review protocol was "reported" in accordance with PRISMA-P.

Point 2: Please note that exclusion criteria are not opposite of inclusion criteria. Therefore, the following should be removed from the manuscript: "Case control, proof-of-concept, two-gate studies and case studies will be excluded"; since this systematic review is on interventional controlled trials.

Response 2: We agree with the editor that clear exclusion criteria needs to be stated and that it is not solely the opposite of inclusion criteria. We removed the following sentence: "Case control, proof-of-concept, two-gate studies and case studies will be excluded." In addition, we created a short paragraph describing exclusion criteria.

Point 3: The authors need to provide adequate and detailed search strategy for at least one of the databases considered for this systematic review. This should be provided as a supplementary file.

Response 3: We apologize for being unclear with our search strategy and for not including a detailed supplementary file. We included a new supplementary file detailing the databases used and the search terms used.

Point 4: The authors declared that "The search strategy will be composed of four concepts combined with Boolean operators: "nature," "chronic pain," "treatment," and "controlled trials." Considering the PICOS framework and the research question in this systematic review, the 'concept' "treatment" does not seem to be the most adequate in the search strategy to denote the 'outcome'.

Response 4: We agree with the editor that the listed Boolean operators are not an adequate search strategy. The aforementioned sentence does not accurately or fully reflect our intended search strategy and was removed from our protocol. We added a sentence describing the use of the PICOS framework, and listed search terms in a search strategy supplementary file.

Point 5: The Editor does not see the added value of a blank flow-chart in a protocol manuscript, though this practice has been sometimes seen in the literature. Please consider removing this.

Response 5: We have previously seen the use of a blank flow-chart in other review protocols, but agree with the editor that it does not serve much purpose. We will remove this from our review.

Point 6: Instead of presenting the following subtitles in the methods section (i.e., 'Types of Studies', 'Types of Participants ', 'Types of Intervention') and in accordance with the PRISMA-P checklist, the Editor advises the authors to report the 'Eligibility criteria', as a clear section, with one paragraph on inclusion criteria, and another one on exclusion criteria. Please consider being concise. Then, another separate section on 'Outcomes and prioritization' should be reported.

Response 6: We followed the above recommendations and re-organized the methods to report 'Eligibility criteria' as a section without subtitles and concise paragraphs describing inclusion criteria and exclusion criteria.

Point 7: The following should be removed from the data extraction section: "Risk of bias: specified under Assessment of risk bias in included studies". This is not a data to extract.

Response 6: As recommended, we removed the following from the data extraction section: "Risk of bias: specified under Assessment of risk bias in included studies" as it is not data.

Point 8: For the structure of the methods section, please consider strictly following the PRISMA-P. This will ease reading and understanding for potential future readers of this manuscript.

Response 8: We agree with the editor that the previous structure of the methods section could potentially be difficult to follow. We restructured the methods section to follow the general structure of the PRISMA-P checklist.

Point 9: For risk of bias assessment, instead of stating "… will independently assess the risk in accordance with the Cochrane Handbook for Systematic Reviews of Interventions", please state that the Cochrane risk of bias tool will be used (not the handbook).

Response 9: We thank the editor for making us aware of this mistake. We agree with the editor that the Cochrane risk of bias tool should be cited instead of the Cochrane handbook. The section was edited to reflect those changes.

Point 10: In accordance with the PRISMA-P, please remove the GRADE assessment from the data analysis section. These are indeed two different things.

Response 10: After discussion with the authors and reviewing the PRISMA-P, we agreed with the editor and removed the GRADE assessment from the data analysis section. The GRADE assessment discussion was moved to the 'Assessing certainty in findings' section.

Point 11: The Data synthesis section is very poorly described. Please refer to previous well reported meta-analyses and to the Cochrane handbook for systematic reviews for guidance. The statistical analyses planned should be clearly reported in this section, with assumptions guiding the choices made.

Response 11: We apologize to the editor that the data synthesis section was poorly described and articulated. We first removed the discussion on the GRADE assessment and moved it to the 'Assessing certainty in findings' section. We then reorganized the section into the following paragraphs: criteria for meta-analysis and mean calculations, subgroup analysis, assessment of heterogeneity and sensitivity analysis. Sentences that were deemed confusing were removed.

Point 12: Please remove the section on "Measures of treatment effect" from the manuscript and move relevant information to the data analysis section. Please note that the following

sentence in this section does not make any sense: “To test the significance of the RR and 95% CI, we will use the chi squared test”.

Response 12: After restructuring the methods section to follow the PRISMA-P, the contents that were previously in ‘Measurements of treatment effect’ were discussed in the ‘Data analysis/synthesis’ section. We agree with the author that the sentence “To test the significance of the RR and 95% CI, we will use the chi squared test” was confusing; hence, was removed. Instead we included the following 2 sentences: “We will present effect size in the form of mean difference (MD) and will calculate their corresponding 95% confidence intervals. In contrast, the effect size for studies with different outcome measurements will be presented using the standardized mean difference (SMD).”

Point 13: What is the meaning of this sentence in a meta-analysis where the authors are planning the calculate SMDs: “T-tests and ANOVA tests will be utilized when comparing two means and multiple mean values respectively”?

Response 13: We agree with the editor that the aforementioned sentence was confusing and unclear. After discussion, the authors decided to remove this sentence as the ‘Data analysis and synthesis’ section discusses the use of mean difference, standardized mean differences and the 95% confidence intervals to determine significance.

Point 14: The Editor advises the authors to take the necessary time to adequately revise this manuscript before resubmitting it to the Journal. If the authors feel it necessary, the Editor advises them to ask for the help of colleagues with strong experience in the conduct of systematic reviews and meta-analyses, to improve this protocol (indeed, the Editor thinks this may be necessary).

Response 14: We thank the editor for taking the time to provide us with the feedback and commentary. The authors have taken the time to re-evaluate the organization of the systematic review protocol and the proposed methodology and have reviewed previous protocol publications. We believe that we have made the necessary adjustments to improve the overall organization and content of this review protocol. We focused on improving the structure of the methods section, while ensuring that each part was detailed, clear and reflected the goals of this review.

Below are the responses to comments provided by <u>Reviewer 1:</u>

Point 1: Greenspace” should be added to search terms in addition to just “nature,” especially given greenspace and the color green may have explicit pain reducing properties (perhaps more so than other types of nature, however that remains to be seen).

Response 1: We thank the reviewer for the suggestion of "greenspace" to our search strategy. In our revised manuscript, is our detailed search strategy which includes many terms in addition to nature, including "greenspace."

Point 2: While this may be the intentions of the authors already, it would help to explicitly state in the data extraction more overt details in addition to "type" of nature. Does this mean virtual or actual nature exposure? Or does this mean various nature ecosystems such as greenspace versus bluespace or others? There are also active versus passive experiences with nature, does this matter? How much time of exposure would also be very helpful, as there may be a minimum threshold. Just a few more explicit details on what the authors intend to collect would be very helpful. A couple of these thoughts came to the fore in the discussion, however should be listed explicitly in the methods.

Response 2: We appreciate the comments provided by the reviewer. We agree that we should explicitly state more overt details in our data extraction section to reflect both the inclusion criteria and the intended subgroup analyses. We added more explicit details to indicate that we intend to collect data concerning the form of intervention (physical vs. virtual), the ecosystem experienced, the type of experience (active vs. passive), the duration of the intervention and follow-up. We additionally reflected this in our description of subgroup analysis.

Point 3: The above point will be a critical component of the discussion of this review. The implications being that perhaps some nature exposure might be beneficial, but there might be a minimum threshold that is needed to reach that point (e.g., minutes spent viewing nature, "quality" of nature viewed). From this exciting review, I hope that the authors are able to glean specific recommendations moving forward for both future research and practice. However if the actual intervention of nature (i.e., the independent variable in this case), is not documented extensively, the review may fall short of this.

Response 3: We thank the reviewer for the aforementioned comments. We agree that given the breadth of nature exposure, there is much data to collect and document to ensure that we can glean recommendations for future research and practices. As mentioned above, we updated our data extraction section to reflect our intent to collect a variety of information concerning the intervention and the participant experience.

Point 4: May also be worth noting that nature prescriptions from physicians are gaining traction in several countries, but specific guidelines for these prescriptions for any conditions (including pain) are not well fleshed out, and this review will be a critical step in synthesizing the research to reach "prescription guidelines."

Response 4: We agree with the reviewer that it would be interesting to include a short discussion about the lack of guidelines concerning nature prescriptions. We updated our discussion to include several sentences describing the lack of specific guidelines and how our review may potentially serve as a resource for physicians in the treatment of chronic pain.

Below are the responses to comments provided by Reviewer 2:

Point 1: In general, the protocol regarding a systematic review and meta-analysis of the effect of nature exposure on pain experience and quality of life in patients with chronic pain was well written. However, no control or comparison was described in the PICO framework. Besides, inclusion and exclusion criteria of the articles were not described in detail.

Response 1: We thank the reviewer for taking the time to read and comment on our review protocol. We agree that there was no clear comparator group described in the protocol. We included a description of the comparator group in our updated 'Eligibility criteria' section. The section is organized in the PICO format. We also agree with the reviewer that initial inclusion and exclusion criteria were not clear. We reorganized the 'Eligibility criteria' to ensure that the inclusion and exclusion criteria are clearly described.

Sincerely,
Matthew J. Lee
Medical Student, Graduating Class of 2025
Hackensack Meridian School of Medicine
