# Supplementary material for: The effect of nature exposure on pain experience and quality of life in patients with chronic pain: A systematic review and meta-analysis protocol
Source: PLoS One. 2023 Sep 28;18(9):e0291053. doi: 10.1371/journal.pone.0291053 (PMC10538778; doi:10.1371/journal.pone.0291053)
Supplement: Attachment — Submitted filename: Response to Reviewers V2.pdf [file pone.0291053.s005.pdf]

Hackensack Meridian
School of Medicine

Dear Editor and Reviewers,

Thank you for your comments and feedback on our manuscript entitled “The Effect of Nature Exposure on Pain Experience and Quality of Life in Patients with Chronic Pain: A Systematic Review and Meta-Analysis Protocol.” We appreciate you taking the time to help improve our systematic review protocol. Our most recent revisions are marked in red in the updated manuscript.

Below are our responses to the following comments provided by the Editor:

Point 1: Abstract: Please remove the following from the abstract, as per a previous Editor’s comment: “Case control, proof-of-concept, two-gate studies and case studies will be excluded” (lines 32-33).

Response 1: We thank the editor for bringing this to our attention. We removed the sentence “Case control, proof-of-concept, two-gate studies and case studies will be excluded” from our abstract to reflect the methods section of our protocol.

Point 2: Introduction to the methods section: Please consider reporting first which methodology guideline will be followed to conduct this systematic review before stating which one was followed to report the current protocol. Please refer to the Editor’s previous comment n°1 (first peer review round). In addition to these two guidelines, please also add that once completed, this systematic review will be reported according to the PRISMA 2020 statement.

Response 2: We agree with the editor's suggestion regarding the sequencing of methodological guidelines and have revised the text accordingly. We describe that we will follow the Cochrane Handbook for Systematic Reviews of Interventions in conducting the systematic review. We then mention the guidelines used to report our protocol (PRISMA-P) followed by the guideline we will use to report the systematic review (PRISMA 2020 statement).

Point 3: Eligibility criteria: Please move the exclusion criterion “Studies written in languages other than English will be excluded” to the end of the list of exclusion criteria, as this is not the most important one in this study.

Response 3: We agree with the suggestion on reordering our exclusion criteria. We have now moved the language criterion to the end of the list to properly reflect its relative importance

Point 4: Section on "Search strategy": Please remove the sentence starting by "To ensure consistency and collaboration between…" (lines 120-122), as this has nothing to do with search strategies.

Response 4: We thank the editor for bringing this to our attention. The sentence mentioned has been removed for better focus and precision in describing our search strategy

Point 5: Please consider providing the search strategies as a supplementary MS Word document. Second, as Medline and Embase do not use the same thesaurus, please note that it is necessary to provide search strategies tailored to each database separately (including CINAHL). Please note that the validity of systematic reviews primarily depends on the quality of search strategies.

Response 5: We appreciate the reviewer's constructive feedback on our search strategies. In response, we provided our search strategies as a supplementary MS Word document. We understand the unique thesaurus requirements of Medline, Embase, and CINAHL and tailored our search strategies separately for each database to ensure a comprehensive and relevant search. We acknowledge the importance of robust search strategies to the validity of systematic reviews and appreciate the guidance provided.

Point 6: Selection of studies: Please change "Studies inconsistent with the inclusion criteria will be excluded" to "Studies inconsistent with the eligibility criteria will be excluded". Please note that both inclusion and exclusion criteria are always considered for studies selection (including title/abstract selection). The last but one sentence (lines 129-131) is not very clear. Please rephrase or delete; then add that full text selection will follow title/abstract selection.

Response 6: We concur with the reviewer's suggestions for improving our study selection process. We have refined the language to specify 'eligibility criteria', and clarified our process for title, abstract, and full text screening.

Point 7: Data analysis: For effect sizes calculations, anticipating that different outcome measurements may have been used, please consider using SMDs only (not MD and SMD). This will ease comparisons of treatment effects.Please note that subgroup analysis is heterogeneity assessment. Please remove "If >10 studies are included in meta-analysis, we will assess for heterogeneity". Please refer to the Cochrane handbook and to high quality systematic reviews for guidance to improve this section. Thank you for your understanding.

Response 7: We appreciate the reviewer's insightful comments. In response, we have chosen to utilize only the standardized mean difference (SMD) for effect sizes, understanding that this will ease comparison of treatment effects. We have also clarified our approach to heterogeneity assessment in our methodology, removing the specific statement regarding the number of studies. We agree that subgroup analysis forms an integral part of heterogeneity assessment and have revised our protocol accordingly, utilizing the Cochrane handbook and high-quality systematic reviews.

Point 8: Please consider changing the title ‘Assessing certainty in findings’ to ‘Assessing the certainly of evidence’.

Response 8: We appreciate the suggestion regarding the title adjustment. We agree and have changed the section title to reflect 'Assessing the certainty of evidence' for clarity and precision.

Sincerely,
Matthew J. Lee
Medical Student, Graduating Class of 2025
Hackensack Meridian School of Medicine
